# Advances in deciphering the interactions between viral proteins of influenza A virus and host cellular proteins

**DOI:** 10.1016/j.cellin.2023.100079

**Published:** 2023-01-31

**Authors:** Li Jiang, Hualan Chen, Chengjun Li

**Affiliations:** State Key Laboratory of Veterinary Biotechnology, Harbin Veterinary Research Institute, Chinese Academy of Agricultural Sciences, Harbin, China

## Abstract

Influenza A virus (IAV) poses a severe threat to the health of animals and humans. The genome of IAV consists of eight single-stranded negative-sense RNA segments, encoding ten essential proteins as well as certain accessory proteins. In the process of virus replication, amino acid substitutions continuously accumulate, and genetic reassortment between virus strains readily occurs. Due to this high genetic variability, new viruses that threaten animal and human health can emerge at any time. Therefore, the study on IAV has always been a focus of veterinary medicine and public health. The replication, pathogenesis, and transmission of IAV involve intricate interplay between the virus and host. On one hand, the entire replication cycle of IAV relies on numerous proviral host proteins that effectively allow the virus to adapt to its host and support its replication. On the other hand, some host proteins play restricting roles at different stages of the viral replication cycle. The mechanisms of interaction between viral proteins and host cellular proteins are currently receiving particular interest in IAV research. In this review, we briefly summarize the current advances in our understanding of the mechanisms by which host proteins affect virus replication, pathogenesis, or transmission by interacting with viral proteins. Such information about the interplay between IAV and host proteins could provide insights into how IAV causes disease and spreads, and might help support the development of antiviral drugs or therapeutic approaches.

## 1 Introduction

Influenza viruses belong to the family Orthomyxoviridae and are categorized into four types, A, B, C, and D. Among the four virus types, influenza A virus (IAV) is a widespread zoonotic pathogen that can infect humans and various animal species. IAV is further classified into different subtypes on the basis of the antigenicity of its two glycoproteins, hemagglutinin (HA) and neuraminidase (NA). In humans, IAV causes seasonal epidemics and occasional pandemics. In birds, the circulation of avian influenza viruses worldwide threatens the poultry industry. Importantly, the wide distribution of avian influenza viruses has also led to spillover to humans. In particular, H5 avian influenza virus has caused devastating damage to the world poultry industry, and has occasionally infected humans, leading to serious illness and death (Cui et al., 2022a, 2022b; Gu et al., 2022; Lee et al., 2021). A novel H7N9 avian influenza virus emerged in 2013 in China, posing a severe risk to human health and the poultry industry (Gao et al., 2013; Shi et al., 2017, 2018; Wan et al., 2022; Zhang et al., 2013).

**Fig. 1:**
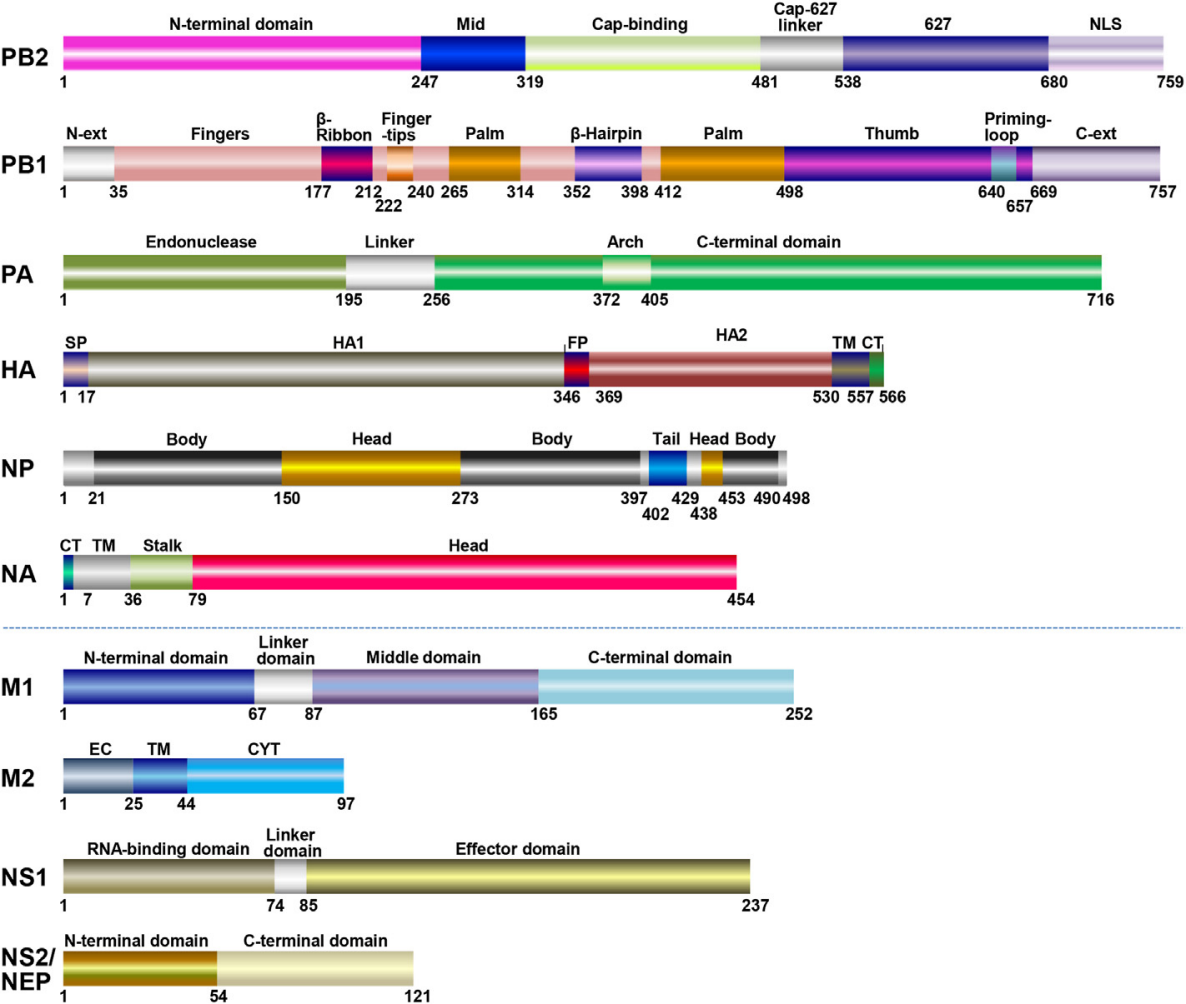
**Schematic representation of ten essential proteins of IAV.** The structural and/or functional domains for PB2, PB1, PA, HA, NP, NA, M1, M2, NS1 and NS2/NEP of IAV are shown, among which, the domains for HA, NA, and NS1 are displayed in the background of A/Udorn/72 (H3N2), A/Puerto Rico/8/34 (H1N1), and A/Udorn/72 (H3N2) virus, respectively. Abbreviations: NLS, nuclear localization signal; ext, extension; SP, signal peptide; FP, fusion peptide; TM, transmembrane domain; CT, cytoplasmic tail; EC, extracellular domain; CYT, cytoplasmic domain.

The genome of IAV encodes up to 18 different viral proteins (Yamayoshi et al., 2016), including 10 essential proteins: polymerase basic protein 2 (PB2), polymerase basic protein 1 (PB1), polymerase acidic protein (PA), HA, nucleoprotein (NP), NA, matrix protein 1 (M1), matrix protein 2 (M2), and nonstructural protein 1 (NS1) and 2 (NS2, also known as nuclear export protein, NEP) (Fig. 1) (Barman et al., 2004; Broecker et al., 2019; Chen et al., 2005, 2020; Fan et al., 2019; Huang et al., 2013; Iwatsuki-Horimoto et al., 2006; Lorieau et al., 2010; Luo et al., 2018; Noton et al., 2007). During its evolution and adaptation to its host, IAV expertly modulates and hijacks the host machinery to complete its replication cycle. These modulations alter host cellular responses and enable the optimal expression of the viral products throughout the infection. Due to limited encoding ability, each viral protein plays a vital role during IAV replication by interacting with various numbers of host cellular factors. In contrast, the host defense system also utilizes host restricting factors to target the viral proteins and antagonize viral replication.

Over the past 20 years, extensive studies on the interplay between IAV and its host have been carried out. The current strategies employed for screening and identifying host factors include, but are not limited to, whole genome siRNA library screening, CRISPR/CAS9 library screening, haploid screening, yeast two-hybrid screening, and GST-pulldown combined with mass spectrometry. In this active research area, a number of novel host factors have been identified and their roles in the replication, pathogenesis, and transmission of IAV well investigated. It is important to point out that host factors involved in the regulation of the IAV life cycle can either interact with the viral proteins, thereby affecting the viral protein function, stability, localization, and transport, or indirectly participate in host cellular processes or signaling pathways that affect virus replication. In this review, we will focus on those host cellular proteins that interact with the essential viral proteins of IAV. Due to the relatively high specificity of such interactions, the knowledge accumulated not only improves the current understanding of the mechanism of virus-host interaction in the course of IAV infection, but also provide a reference for the development of effective countermeasures, such as antiviral drugs.

## 2 Main body

### 2.1 Host proteins that interact with the viral polymerase proteins

The RNA-dependent RNA polymerase of IAV is responsible for the transcription and replication of the viral genome and consists of three subunits: PB2, PB1, and PA. The PB1 protein is the core of the polymerase complex; it binds to the PA protein through its N-terminus, whereas its C-terminus binds to the PB2 protein (Gonzalez et al., 1996). The three polymerase proteins assemble into a complex in the nucleus. PB2 functions to bind to the 5' cap structure of host mRNA precursors, which is an important step in generating the primers required for viral mRNA transcription (Blaas et al., 1982). PB1 is the core enzyme in catalyzing viral genome transcription and replication by binding to the ends of viral RNA (vRNA) (Li et al., 1998). PA uses its endonuclease activity to cleave the 5' cap structure of the host mRNA precursors, thereby completing the cap-snatching process (Li et al., 2001).

Several host factors promote IAV replication by interacting with viral polymerase proteins. Cytosolic chaperonin containing TCP-1 (CCT) interacts with PB2 as a monomer, and ensures the expression and possibly the folding of PB2 during IAV infection (Fislova et al., 2010). Three components of CUL4-RING E3 ubiquitin ligases (CRL4s)—DDB1 adaptor and the two substrate recognition receptors DCAF12L1 and DCAF11—interact with PB2 and mediate K29-linked ubiquitination of PB2, which promotes the replication of IAV (Karim et al., 2020). In the late stage of the viral life cycle, the switch I region of Rab11a interacts with the C-terminus of PB2, thereby facilitating the trafficking of the viral ribonucleoprotein (vRNP) complex along the outward transport route for assembly (Veler et al., 2022). The nuclear import of the PB1-PA heterodimer is mediated by the interaction between PB1 and Ran-binding protein 5 (RanBP5). Mutations in PB1 that disrupt RanBP5-binding reduce the nuclear accumulation of the PB1-PA heterodimer and severely attenuate virus replication (Hutchinson et al., 2011).

The three viral polymerase proteins are also targets of host restricting factors. Tu elongation factor, mitochondrial (TUFM), exhibits a higher binding affinity for PB2-627E than for PB2-627K in human cells. It appears that TUFM interacts with PB2-627E in mitochondria, which suppresses the replication of PB2-627E virus in an autophagy-dependent manner (Kuo et al., 2017). Tripartite motif containing 32 (TRIM32) interacts with PB1, and translocates with it into the nucleus during IAV infection. The ubiquitin E3 ligase activity of TRIM32 directly ubiquitinates PB1, leading to PB1 degradation and consequent reductions in viral polymerase activity and growth titer (Fu et al., 2015). Plakophilin 2 (PKP2) interacts with PB1, which then interferes with the interaction between PB2 and PB1, thereby inhibiting viral polymerase activity and virus replication (Wang et al., 2017). HCLS1-associated protein X-1 (HAX1), an antiapoptotic host protein, interacts with the nuclear localization signal (NLS) domain of PA, and impedes the nuclear import of PA, thereby suppressing viral polymerase activity and virus replication (Hsu et al., 2013).

In the meantime, the polymerase proteins of IAV interact extensively with the host innate immune system. They all bind to retinoic acid-inducible gene I (RIG-I) in an RNA-independent manner (Li et al., 2014), and vRNP complexes bearing PB2-627E are more prone to recognition and restriction by RIG-I than those bearing PB2-627K (Weber et al., 2015). They also interact with mitochondrial antiviral-signaling protein (MAVS) and suppress the activation of the MAVS-mediated type I interferon (IFN) pathway (Iwai et al., 2010). The PB1 of H7N9 viruses is preferentially associated with a selective autophagic receptor neighbor of BRCA1 (NBR1) that recognizes ubiquitinated MAVS and delivers it to autophagosomes for degradation (Zeng et al., 2021). Of note, an interaction circuit is formed among TRIM35, TNF receptor associated factor 3 (TRAF3), and PB2 during IAV infection. PB2 inhibits K63-linked ubiquitination of TRAF3 and the consequent activation of RIG-I antiviral signaling, whereas TRIM35 defends the host against IAV infection by catalyzing K63-linked polyubiquitination of TRAF3 and activating RIG-I antiviral signaling, and by directly mediating K48-linked polyubiquitination and degradation of PB2 (Sun, Jiang, et al., 2020). Moreover, PB2 mediates polyubiquitination and degradation of Janus kinase 1 (JAK1) at lysines 859 and 860, which contributes to viral replication in mammalian cells and pathogenicity in mice (Yang et al., 2022).

### 2.2 Host proteins that interact with the NP protein

NP is a major structural protein of IAV, and an important component of the vRNP complex. It possesses two NLSs that determine its localization in the nucleus of infected cells: one is located between amino acids 3–13 at the N terminus of NP, and is an unconventional NLS (Wang et al., 1997); the other is a bipartite NLS that sits between amino acids 198 and 216 (Weber et al., 1998).

During IAV replication, NP protein acts as viral RNA carrier in the vRNP complex. Tat specific factor 1 (Tat-SF1) interacts with free NP, but not with NP associated with RNA, and facilitates the formation of RNA-NP complexes, thereby acting as a molecular chaperone for NP (Naito et al., 2007a). Pre-mRNA processing factor 18 (Prp18) also functions as a chaperone for NP, promoting the formation of NP-RNA complexes and facilitating the dissociation of newly synthesized RNA from the template after the early elongation step to stimulate the elongation reaction (Minakuchi et al., 2017). DEAD-box RNA helicases DDX39A and DDX39B interact with NP, and modulate the oligomerization state of NP, thereby finely regulating the activity of vRNP complex (Zhao et al., 2022). Fragile X Mental Retardation Protein (FMRP) interacts with NP to facilitate the assembly and nuclear export of the vRNP complex, which is important for the replication of neurovirulent avian influenza viruses in neuronal cells (Zhang et al., 2021). High-mobility-group box (HMGB) protein 1 (HMGB1) interacts with NP in the nucleus, and enhances vRNP complex activity and virus replication (Moisy et al., 2012). It has been proposed that the interaction between NP and HMGB1 may facilitate the recruitment of vRNPs at transcriptionally active sites of chromatin, which is critical for the transcription/replication of the IAV genome. Moreover, nuclear factor 90 (NF90) interacts with NP in the nucleus, and inhibits both transcription and replication of viral RNA (Wang et al., 2009).

In the early stage of virus infection, the vRNP complex enters the nucleus to initiate transcription and replication of the viral genomic RNA. The interaction between the NLS of NP and isoforms of importin α family members further recruits importin β, which mediates the nuclear import of NP and the vRNP complex. During this process, Bcl10-interacting protein with CARD 1 (BinCARD1) binds to NP and specifically enhances the interaction between NP and importin α7, thus facilitating the nuclear import of the vRNP complex and newly synthesize NP (Wang et al., 2022a). In contrast, moloney leukemia virus 10 (MOV10) interacts with NP and disrupts the binding of NP to importin α, resulting in the retention of NP in the cytoplasm and the consequent impairment of the vRNP complex function (Zhang et al., 2016). Phospholipid scramblase 1 (PLSCR1) forms a trimeric complex with NP and importin α, which inhibits the incorporation of importin β into the complex, thus impairing the nuclear import of NP and suppressing virus replication (Luo et al., 2018).

Of note, the function of NP is extensively regulated by the host posttranslational modification system. In particular, TRIM family proteins interact with NP to antagonize IAV replication. For example, TRIM22 and TRIM41 interact with NP, and act as E3 ubiquitin ligases to mediate the polyubiquitination and proteasomal degradation of NP (Di Pietro et al., 2013; Patil et al., 2018).

### 2.3 Host proteins that interact with HA

HA is one of the two glycoproteins encoded by IAV genome. The precursor of HA, HA0, is cleaved by protease to produce HA1 and HA2, thus enabling HA to fulfill its function in virus infection. The mature HA protein is a trimeric type I transmembrane protein, with its C-terminus inserted into the viral envelope and its hydrophilic N-terminus protruding from the virion surface to form spikes. The first major function of HA is to bind to the sialic acid receptors on the surface of host cells, and the receptor binding site of HA is located in its globular head. The second major function of HA is to induce fusion between the viral envelope and the endosome membrane under acidic conditions, which is required for the viral uncoating process.

Pertaining to its function in the entry process, HA interacts with transmembrane protein immunoglobulin superfamily DCC subclass member 4 (IGDCC4), which plays an important role in the internalization of IAV into host cells. In vivo studies indicate that the replication and virulence of H5N1 virus in IGDCC4-knockout mice is significantly reduced compared with that in wild-type mice (Song et al., 2021). Similarly, HA interacts with free fatty acid receptor 2 (FFAR2) on the surface of infected cells, which facilitates the internalization of IAV. Further studies have shown that the FFAR2-β-arrestin1-AP2B1 signaling cascade is important for the efficient internalization of IAV during the entry stage (Wang et al., 2020).

The cleavage of HA by host proteases is essential for IAV infectivity. The HAs of human H7N9 and H1N1 viruses are efficiently cleaved by transmembrane serine protease 2 (TMPRSS2), whereas the HA of H3N2 virus is less susceptible to TMPRSS2. Consistently, the replication of H7N9 and H1N1 viruses is significantly inhibited in TMPRSS2-knockout mice (Hatesuer et al., 2013; Tarnow et al., 2014). Similarly, matriptase, a member of the type II transmembrane serine protease family, can cleave and activate the HA of H1N1 virus but not that of H2N2 or H3N2 viruses (Hamilton et al., 2012). By contrast, both TMPRSS2 and TMPRSS4 are required for the efficient cleavage of HA and spread in mouse lung of H3N2 virus (Kuhn et al., 2016). TMPRSS11A, a TMPRSS2-related enzyme, can also cleave and activate the HA of H1N1 and H3N2 viruses, and increase virus dissemination in cell culture (Zmora et al., 2018).

### 2.4 Host proteins that interact with NA

NA is the other glycoprotein on the surface of influenza virions, and is a typical type II glycoprotein. NA assembles as a tetramer structurally, and each monomer consists of a short cytoplasmic tail, a transmembrane region, a stalk, and a catalytic head. It cleaves sialic acid on the surface of host cells and progeny virions, thereby promoting virus budding and release.

Transforming growth factor-beta (TGF-β) plays multiple roles in immune regulation by controlling inflammatory responses. During IAV infection, NA activates latent TGF-β (LTGF-β) by removing its sialic acid motifs, thus converting biologically inactive LTGF-β to its active form TGF-β. Activated TGF-β plays a pivotal role in protecting the host from influenza (Carlson et al., 2010). Similarly, cluster of differentiation 83 (CD83) is a sialylated glycoprotein expressed on the surface of dendritic cells and macrophages in the lung. NA targets CD83, modifies its status on the cell surface, and enhances its role in the induction of cytokine production, thereby contributing to the cytokine storm during IAV infection (Ma et al., 2021). In addition, host membrane glycoprotein carcinoembryonic antigen-related cell adhesion molecule 6 (CEACAM6) interacts with NA, and enhances host cell survival by activating the Src/Akt signaling axis, which facilitates the replication of IAV (Gaur et al., 2012).

### 2.5 Host proteins that interact with the M1 protein

M1 is the most abundant structural protein of IAV. It shuttles between the nucleus and cytoplasm and plays an important role in the life cycle of IAV. In the nucleus, M1 binds to the vRNP complex and NS2/NEP protein, and NS2/NEP protein binds to CRM1, which leads to the nuclear export of the vRNP complex (Paterson & Fodor, 2012). M1 is also a driving force for virus packaging and morphogenesis. It directly interacts with the cytoplasmic regions of the HA, NA, and M2 proteins as well as the vRNP complex, thus acting as a bridge between the inner core components and the outer envelope viral proteins (Nayak et al., 2009).

Several host cellular proteins are involved in the life cycle of IAV by interacting with M1. In the uncoating process, histone deacetylase 6 (HDAC6) interacts with M1, which releases the M1 shell from the endosome surface and ruptures it, resulting in the release of the eight bundled vRNPs into the cytosol (Banerjee et al., 2014). Transportin 1 (TNPO1), a member of the importin β family, binds to the NLS sequence motif of M1 close to the N terminus and removes residual M1 from the vRNP surface (Miyake et al., 2019). The action of TNPO1 leads to the dissociation of vRNPs from each other, which are then translocated into the nucleus via the classical nuclear import pathway. In the late stage of IAV replication, proteasome 26S subunit, non-ATPase 12 (PSMD12) interacts with M1 and promotes the replication of IAV (Hui et al., 2022). Mechanistically, PSMD12-mediated K63-linked ubiquitination of M1 at the K102 site is essential for the morphogenesis and budding of virus particles. M1 also binds to G protein subunit β1 (GNB1), which facilitates M1 transport to budding sites and promotes the release of progeny viruses (Li et al., 2021).

### 2.6 Host proteins that interact with the M2 protein

M2 protein is translated from a spliced mRNA transcript of vRNA segment 7 of IAV. It is 97 amino acids in length and comprises three distinct domains: a 24-residue ectodomain, a 19-residue transmembrane domain, and a 54-residue cytoplasmic tail (CT) domain (Pinto et al., 1992). In the early stage of viral replication, the ion channel activity of M2 is essential to dissociate the vRNP complex from M1 and the lipid bilayers, thus completing the uncoating process (Pinto & Lamb, 2006). M2 is positioned at the edge of the budozone when virus budding begins; during virus release, M2 locates to the neck of the budding virion and ultimately pinches it off (Roberts et al., 2013).

In the late stage of the IAV life cycle, several host cellular proteins affect viral replication by interacting with M2. The interaction of M2 with transport protein particle complex 6A delta (TRAPPC6AΔ) is mediated by a highly conserved leucine residue at position 96 of M2. This interaction regulates the trafficking of M2 to the apical plasma membrane, favors viral replication in vitro, and positively modulates virus virulence in mice (Zhu et al., 2017). Cyclin D3 binds to M2 and interferes with the interaction between M2 and M1, thus impairing the assembly of progeny virions and attenuating virus replication (Fan et al., 2017). During virus budding and release, membrane-associated RING-CH protein 8 (MARCH8), an E3 ubiquitin ligase, interacts with M2 and mediates its K63-linked polyubiquitination at the K78 residue, resulting in the redirection of M2 from the plasma membrane to the lysosome for degradation (Liu, Xu, et al., 2021). Annexin A6 (AnxA6) protein interacts with M2 and impairs the budding and release of infectious virus particles (Ma et al., 2012).

M2 has been shown to interact with the autophagy regulator TBC1 domain family member 5 (TBC1D5), which binds to Rab7 to enable fusion of autophagosomes and lysosomes. The binding between M2 and TBC1D5 abolishes the TBC1D5–Rab7 interaction, thereby allowing M2 to evade autophagic degradation in lysosomes and traffic to the virus budding site at the plasma membrane (Martin-Sancho et al., 2021). M2 also interacts with microtubule-associated protein light chain 3 (LC3), redirecting it to the unexpected destination of the plasma membrane, thereby subverting the autophagy process and enabling IAV to evade autophagy restriction. Accordingly, an IAV mutant bearing an LC3-interacting motif mutation in M2 is impaired in virus budding and virion stability (Beale et al., 2014).

The interaction between M2 and host cellular proteins is also involved in the regulation of the innate immune response. M2 interacts with MAVS and prevents its degradation via the autophagy pathway, thereby allowing MAVS to play its role in the activation of antiviral innate immunity (Wang, Zhu, Lin, et al., 2019). P58IPK (58-kDa inhibitor of the interferon-induced double-stranded RNA-activated protein kinase), an inhibitor of protein kinase R (PKR), forms a complex with heat shock protein 40 (HSP40) and M2, which lifts the restriction of P58IPK on the autophosphorylation of PKR. Consequently, the activated PKR functions to restrict IAV replication (Guan et al., 2010). Furthermore, M2 interacts with G protein pathway suppressor 1 (GPS1), which activates the nuclear factor kappa-B (NF-κB) signaling pathway. The activation of the NF-κB signaling pathway plays an important role in the viral polymerase activity of IAV. For this reason, downregulation of GPS1 expression dramatically impairs IAV replication (Kuwahara et al., 2019).

### 2.7 Host proteins that interact with the NS1 protein

The NS1 protein is 202–237 amino acids in length (depending on the virus strain) and consists of two functional domains: the N-terminal RNA-binding domain (RBD) and the C-terminal effector domain (ED) (Engel, 2013). The C terminus of NS1 has a PDZ domain binding motif. The highly pathogenic H5 viruses typically bear the avian virus-type PDZ domain-binding motif ESEV, which mediates binding to a variety of host proteins that contain PDZ domains, thus constituting a major determinant for virus virulence (Fan et al., 2013; Jackson et al., 2008). For instance, the NS1 ESEV PDZ binding motif associates with discs large homologue 1 (Dlg1) and Scribble to disrupt cellular tight junctions, which contribute to the disease severity of H5N1 avian influenza virus infection (Golebiewski et al., 2011).

The most important role of NS1 is to antagonize the host innate immune response, which is achieved through different functions, thereby facilitating virus replication. First, NS1 targets key adaptors in the RIG-I innate immune signaling pathway. It directly binds to RIG-I (Jureka et al., 2020; Mibayashi et al., 2007), or binds to the E3 ubiquitin ligase TRIM25 and blocks TRIM25-mediated ubiquitination of RIG-I (Gack et al., 2009), thus suppressing the activation of RIG-I signaling pathway and inhibiting IFN production. It contains a conserved FTEE motif at positions 150–153, where the E152/E153 residues bind to TRAF3 to block TRAF3 ubiquitination and type I IFN production (Lin et al., 2021). It interacts with protein activator of the interferon-induced protein kinase (PACT), which impairs the association between PACT and RIG-I, and consequently inhibits RIG-I-mediated type I IFN production (Tawaratsumida et al., 2014). It associates with host cellular decay factor 5'-3' exoribonuclease 1 (XRN1) in cellular processing bodies (P-bodies), thereby promoting IAV replication by suppressing RIG-I-mediated type I IFN production (Liu, Mok, et al., 2021). Second, NS1 interacts with the C-terminal domain of YAP/TAZ (Yes-associated protein 1/transcriptional coactivator with PDZ-binding motif) and facilitates their nuclear localization, which blocks toll like receptor 3 (TLR3)-mediated antiviral innate immune signaling via downregulation of TLR3 expression (Zhang et al., 2022). Third, NS1 protein can form a complex with PKR and inhibit PKR activation induced by double-stranded RNA or PACT, thereby inhibiting the PKR-mediated antiviral response (Li et al., 2006).

NS1 contributes to IAV virulence by activating the phosphoinositide 3-kinase (PI3K) signaling pathway. The NS1 protein binds to the P85β subunit of PI3K to activate the PI3K/Akt signaling pathway and inhibit apoptosis, thus promoting the growth of IAV (Hale et al., 2006, 2008; Shin et al., 2007). The NS1 protein of 1918 H1N1 virus and many avian influenza viruses can bind to host proteins containing SH3 domains, such as Crk/CrkL (CT10 regulator of kinase/Crk-like protein), which can also promote activation of PI3K signaling and facilitate viral replication (Heikkinen et al., 2008).

NS1 is an expert in modulating the expression of viral and host proteins. It specifically enhances the translation of viral but not cellular mRNAs. It binds to poly(A) binding protein 1 (PABP1) and eukaryotic translation initiation factor 4GI (eIF4GI), which recruits the 43S pre-translation initiation complex to the viral mRNAs, thereby stimulating the translation of the viral mRNAs (Burgui et al., 2003). Of note, NS1 interacts with the cellular NS1-binding protein (NS1-BP) to promote splicing and nuclear export of M mRNAs (Zhang et al., 2018). Moreover, the splicing factor 2/alternative splicing factor (SF2/ASF) interacts with an exonic splicing enhancer (ESE) motif in the NEP/NS2 mRNA. NS1 directly interacts with SF2/ASF in the nucleus and suppresses splicing of NEP/NS2 mRNA, thereby maintaining the optimized balance of NEP/NS2 and NS1 protein levels during IAV infection (Huang et al., 2017). Regarding the effect of NS1 on host protein expression, it has been shown to bind to cleavage and polyadenylation specificity factor 30 (CPSF30) and poly(A)-binding protein II (PABII), leading to the inhibition of the 3'-end processing of cellular pre-mRNAs, which plays a critical role in the virulence of IAV (Chen et al., 1999; Twu et al., 2006). NS1 can also prevent the binding of nuclear RNA export factor 1-nuclear transport factor 2-related export protein 1 (NXF1-NXT1) to nucleoporins, thereby inhibiting host mRNA export through the nuclear pore complex into the cytoplasm for translation, especially those mRNAs encoding immune factors (Zhang et al., 2019). Moreover, the RBD of NS1 binds to adenosine deaminase acting on RNA 1 (ADAR1) and enhances its RNA editing activity, which promotes optimal viral protein synthesis and replication (de Chassey et al., 2013).

Finally, NS1 affects the replication and virulence of IAV through other biological mechanisms. It interacts with RNA helicase A (RHA) in an RNA-dependent manner, leading to enhanced viral RNA replication and transcription (Lin et al., 2012). It uses its double-stranded RNA binding activity, mediated by the two key residues R38 and K41 in the RBD, to interact with DExH-box helicase 30 (DHX30) and antagonize its antiviral activity (Chen et al., 2020). It binds to RNA-associated protein 55 kDa (RAP55), one of the components of P-bodies and stress granules, and suppresses the restriction on virus replication imposed by RAP55-associated P-bodies/stress granules (Mok et al., 2012). It interacts with cleavage and polyadenylation specific factor 4 (CPSF4) and modulates its function in the splicing of *TP53*, which suppresses the transcriptional activity of tumor protein p53 and p53-mediated antiviral responses (Dubois et al., 2019). It also associates with DNA methyltransferase 3B (DNMT3B), leading to the dissociation of DNMT3B from the promoters of genes encoding some key regulators of JAK-STAT signaling. This association further changes the localization of DNMT3B from the nucleus to the cytosol, resulting in K48-linked ubiquitination and degradation of DNMT3B in the cytosol (Liu et al., 2019).

### 2.8 Host proteins that interact with the NS2/NEP protein

NS2/NEP, which is 121 amino acids in length, is translated from the spliced mRNA encoded by vRNA segment 8 of IAV. It synergistically cooperates with the M1 protein to mediate the nuclear export of the vRNP complex in the late stage of viral replication (Paterson & Fodor, 2012). The N terminus of NEP/NS2 has two NESs, which are located between amino acids 12–21 and 31–40, and both NESs interact with CRM1 to mediate the nuclear export of the vRNP complex (Huang et al., 2013).

NS2/NEP binds to host aminoacyl-tRNA synthetase-interacting multi-functional protein 2 (AIMP2), protecting it from ubiquitin-mediated degradation. AIMP2 in turn inhibits the ubiquitination of the K242 residue of the M1 protein and instead promote its SUMOylation, thereby enhancing the stability of M1 and promoting M1-mediated nuclear export of the vRNP complex to increase virus replication (Gao et al., 2015). NS2/NEP also interacts with human immunodeficiency virus (HIV) Rev-binding protein (HRB) in the perinuclear region, thus facilitating the transfer of vRNPs from nuclear export to cytoplasmic trafficking complexes (Eisfeld et al., 2011). In addition, NEP/NS2 interacts with the β subunit of F1Fo-ATPase. By recruiting F1Fo in the cytoplasm and employing the ATPase activity of F1Fo, NEP/NS2, bound to the vRNP complex, promotes genome packaging and progeny virion release (Gorai et al., 2012).

### 2.9 Host proteins that interact with multiple viral proteins

Due to the central role of the vRNP complex in the replication cycle of IAV, numerous host cellular proteins simultaneously interact with multiple viral proteins to affect the nuclear import and assembly of the vRNP complex, the transcription and replication activity, and the export of the vRNP complex. Regarding the nuclear import and assembly of the vRNP complex, GNB1 has been shown to interact with PB2, PB1, and PA, which facilitates the nuclear import of PB2 by enhancing the association of PB2 with importin α3, α5, and α7, and also promotes the viral polymerase assembly (Zheng et al., 2022). HSP90 interacts with PB2 and PB1, and translocates into the nucleus of infected cells during IAV infection, which most likely functions as a chaperone to facilitate the formation of the polymerase complex (Momose et al., 2002; Naito et al., 2007b). Activated protein kinase C (PKC) family member PKCδ interacts with PB2, which stabilizes the interaction between PKCδ and NP, leading to enhancement of NP phosphorylation and consequent assembly of NP with newly synthesized vRNA into vRNPs (Mondal et al., 2017). In contrast, several host proteins impair the nuclear import and assembly of the vRNP complex. Among them, SERTA domain containing 3 (SERTAD3) interacts with all three subunits of the viral polymerase, and impairs the formation of the viral polymerase complex and virus replication (Sun, Li, et al., 2020). Myxovirus resistance 1 (Mx1), a type I and type III IFN-induced effector protein with intrinsic antiviral activity, interacts with NP and PB2, and interferes with the assembly of the vRNP complex by inhibiting the interaction between NP and PB2 (Verhelst et al., 2012). Eukaryotic translation elongation factor 1 delta (eEF1D) interacts with all four vRNP proteins in an RNA-dependent manner, and impairs the interaction between NP and importin α5 and the interaction between PB1 and RanBP5, thereby impeding the nuclear import of NP and the PA-PB1 heterodimer and suppressing vRNP assembly (Gao et al., 2020). In addition, DExD-box helicase 21 (DDX21) binds to PB1 and inhibits polymerase assembly, resulting in reduced viral RNA and protein synthesis. However, NS1 overcomes this restriction by binding to DDX21 and displacing PB1 (Chen et al., 2014).

With respect to the transcription and/or replication activity of the viral polymerase, ribosomal RNA processing 1 homolog B (RRP1B) interacts with PB2 and PB1 and facilitates the binding of the viral polymerase with capped cellular mRNA, which is critical for the synthesis of viral mRNA (Su et al., 2015). MORC family CW-type zinc finger 3 (MORC3, also known as nuclear matrix protein 2, NXP2), a member of the microrchidia (MORC) family, interacts with PB1 and PA, and facilitates IAV propagation by enhancing viral mRNA transcription but not vRNA replication (Ver et al., 2015). Phosphatase 6 (PP6) interacts with PB1 and PB2, and promotes viral RNA synthesis (York et al., 2014). During IAV replication, DnaJA1/HSP40 translocates into the nucleus from the cytoplasm, interacts with PB2 and PA, and enhances viral polymerase activity (Cao et al., 2014). In contrast, protein inhibitor of activated STAT 1 (PIAS1) interacts with PB2, PB1 and NP, and inhibits the vRNP complex activity and virus replication in vitro and in vivo. Mechanistically, PIAS1catalyzes robust SUMOylation of PB2 with its SUMO E3 ligase activity, which remarkably reduces the stability of PB2 (Wang et al., 2022b). In addition, TRIM25 inhibits viral RNA synthesis by binding to vRNPs. It also prohibits the movement of RNA into the polymerase complex, thus inhibiting the onset of RNA chain elongation (Meyerson et al., 2017).

Several studies have been conducted to elucidate the role of host factors acidic nuclear phosphoprotein 32 family member A (ANP32A) and B (ANP32B) in the host adaptation and pathogenicity of IAV. ANP32 proteins are key host factors responsible for the restricted polymerase activity of avian influenza viruses in mammalian cells (Long et al., 2016). ANP32 proteins from birds and mammals directly bind trimeric polymerase in the cell nucleus. In birds, such as chickens and ducks, an exon duplication allows the expression of an alternatively spliced, 33 amino acid-longer isoform of ANP32A that effectively supports the viral polymerase activity of avian influenza viruses (Long et al., 2016). These extra 33 amino acids of chicken ANP32A and the PB2 627 domain of the viral polymerase complex both contribute to the enhanced interaction between chicken ANP32A and the viral polymerase (Mistry et al., 2020). Detailed analysis indicates that avian species express three ANP32A splice variants that differ in the composition of the critical insert. The ANP32A variant that lacks the insert is compromised in its support of avian-like IAV replication, and drive selection of mammalian-adaptive mutations in the viral polymerase (Baker et al., 2018; Domingues et al., 2019). Of note, a unique hydrophobic stretch of amino acids in chicken ANP32A that resembles a SUMO interaction motif-like sequence promotes stronger interactions between chicken ANP32A and the viral polymerase (Domingues & Hale, 2017). Different avian influenza viruses differ in their dependence on human ANP32A. The polymerase activity of a 2013 H7N9 virus is more vulnerable to the deficiency of human ANP32A expression compared with a 1998 H9N2 virus, and the H7N9 virus lost the ability to acquire the PB2 E627K mutation during replication in *Anp32a* knockout mice (Liang et al., 2019). Swine ANP32A can support the polymerase activity and replication of avian-origin influenza viruses, which is attributed to a pair of mutations (I106V and P156S) in the leucine-rich repeat and central domains that enhance the binding of ANP32A to the trimeric polymerase of IAV (Peacock et al., 2020; Zhang et al., 2020).

As for the nuclear export of the vRNP complex, clustered mitochondria homologue (CLUH) is transported into the nucleoplasm and SC35-positive speckles by interacting with PB2 and M1, respectively. The depletion of CLUH inhibits the translocation of M1 to SC35-positive speckles with no effect on PB2 localization to the nucleoplasm, which disrupts the subnuclear transport of vRNP, and abolishes vRNP nuclear export (Ando et al., 2016).

Complex interactions among multiple viral proteins and host proteins also have effects on the virus life cycle. NP and M2 interact with LC3, leading to the induction of the AKT-mTOR-dependent autophagy pathway and an increase in heat shock protein 90 alpha family class A member 1 (HSP90AA1) expression. The interaction between PB2 and HSP90AA1 increases viral RNA synthesis, whereas the NP-LC3 and M2-LC3 interactions facilitate the nuclear export of the vRNP complex and the production of progeny viruses, respectively (Wang, Zhu, Zhao, et al., 2019). DEAD-box helicase 3 (DDX3), a host factor involved in the regulation of stress granules (SGs), interacts with NS1 and NP. In the absence of functional NS1, the interaction between DDX3 and NP traps NP in the SGs, leading to impaired virus growth. By contrast, the presence of functional NS1 in wild-type virus blocks the formation of SGs, and the antiviral effect of DDX3 is no longer observed (Raman et al., 2016).

It should be pointed out that several host cellular proteins mentioned in this article, such as RIG-I, MAVS, TRAF3, LC3, TRIM25, and HSP40, interact with multiple viral proteins of IAV. For instance, the three viral polymerases interact with MAVS and suppress the activation of the MAVS-mediated type I IFN pathway (Iwai et al., 2010), whereas M2 interacts with MAVS and prevents its autophagic degradation, thereby allowing it to mediate antiviral innate immune signaling (Wang, Zhu, Lin, et al., 2019). To logically outline the functions of these host cellular proteins in the replication cycle of IAV, they are also often described in the section of individual viral proteins.

## 3 Concluding remarks and future perspectives

**Fig. 2:**
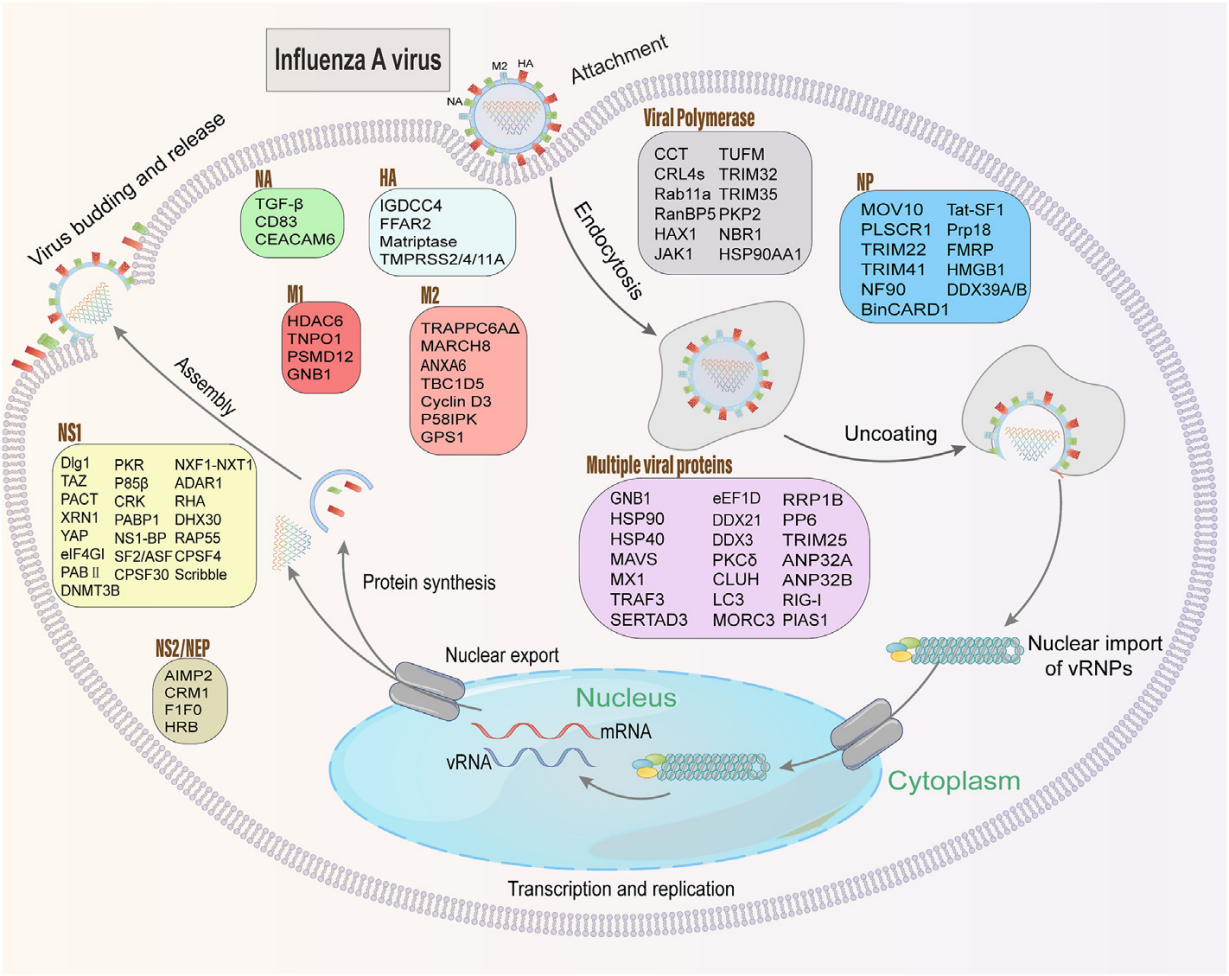
Schematic representation of host cellular proteins that interact with the essential viral proteins of IAV. Host proteins that have been well explored for their roles in the replication cycle of IAV are listed. They are divided into two types: those that interact with the 10 essential viral proteins (i.e., the three polymerase proteins, NP, HA, NA, M1, M2, NS1, and NS2/NEP), and those that simultaneously interact with multiple viral proteins.

As basic research on influenza viruses has advanced, many interacting host factors have been and continue to be identified. In addition to the host cellular proteins reviewed here (Fig. 2), host noncoding RNA is also involved in the regulation of IAV replication (Qu et al., 2021). Of note, the effect of viral and host protein interactions on virus and/or host may stem from distinct mechanisms, such as affecting the expression, localization, stability or transport of proteins, the formation of functional complexes, and the induction or suppression of signaling pathways. In particular, the posttranslational modifications of viral or host proteins function frequently in the process of viral and host protein interactions (Table 1).

**Table 1:** The role of posttranslational modifications (PTMs) in viral and host protein interactions.

Host protein	Interacting viral protein	Role of PTMs in viral and host protein interactions
CRL4s	PB2	Mediates K29-linked ubiquitination of PB2 and promotes virus replication
TRIM35	PB2	Mediates K48-linked polyubiquitination and degradation of PB2
TRAF3	PB2	Inhibits K63-linked ubiquitination of TRAF3 and the consequent activation of RIG-I antiviral signaling
JAK1	PB2	Mediates polyubiquitination and degradation of JAK1, which contributes to virus replication
TRIM32	PB1	Mediates PB1 ubiquitination and degradation, and reduces virus replication
NBR1	PB1	PB1 interacts with NBR1, which promotes the degradation of ubiquitinated MAVS and consequently impairs innate immune signaling
TRIM22	NP	Mediates polyubiquitination and proteasomal degradation of NP
TRIM41	NP	Mediates polyubiquitination and proteasomal degradation of NP
PSMD12	M1	Mediates K63-linked ubiquitination of M1 at the K102 site, which facilities the morphogenesis and budding of virus particles
MARCH8	M2	Mediates K63-linked polyubiquitination at the K78 residue of M2, resulting in its redirection from plasma membrane to lysosome for degradation
P58IPK	M2	The complex formed among P58IPK, HSP40 and M2 lifts the restriction of P58IPK on the autophosphorylation of PKR and PKR-mediated restriction on virus replication
TRIM25	NS1	Blocks TRIM25-mediated ubiquitination of RIG-I, thus suppressing the activation of RIG-I signaling pathway and inhibiting IFN production
TRAF3	NS1	Blocks TRAF3 ubiquitination and type I IFN production
DNMT3B	NS1	Changes the localization of DNMT3B from nucleus to cytosol, followed by K48-linked ubiquitination and degradation in cytosol
AIMP2	NS2/NEP	Protects AIMP2 from ubiquitin-mediated degradation, which subsequently inhibits the ubiquitination of M1 and instead promotes its SUMOylation, thereby enhancing the stability of M1 and promoting M1-mediated nuclear export of the vRNP complex
PKCδ	PB2, NP	Enhances NP phosphorylation and consequent assembly of NP with newly synthesized vRNA into vRNPs
PIAS1	PB2, PB1, NP	Catalyzes robust SUMOylation of PB2 with its SUMO E3 ligase activity, which remarkably reduces the stability of PB2

It should also be noted that the host cellular proteins currently known were identified in different studies. In these studies, the roles of the host proteins were evaluated in different backgrounds, such as cell types, virus strains, and experimental setups. Further studies might be necessary to compare the activity of the host proteins currently identified in parallel under the same experimental conditions, which would be helpful to evaluate their relative importance in the virus replication cycle and identify the ones with potential value in the development of antiviral countermeasures, thereby eventually resolving the drug-resistance issue of current available anti-influenza drugs that all target viral proteins. The ideal host proteins for antiviral drug development would be those that are required for IAV replication, but whose depletion or conditional downregulation by drugs would have no or minimal adverse effects. Furthermore, synergistic effects of different host proteins on the replication of IAV should be tested. The information gained could be useful for the development of cocktail therapies as combinations of antiviral drugs targeting different key host proteins may produce synergistic effects in the prophylaxis and treatment of influenza. In addition, the insights obtained on viral and host protein interactions may also provide clues for the development of more effective vaccines for the prevention and control of influenza. For example, modifying the interaction site of viral and host proteins may enhance the replication of vaccine seed virus and increase the expression level of protective antigens.

The interplay between viral proteins and host cellular proteins is an active area of influenza research. Here, we endeavored to offer a balanced overview of this active research area. Due to space limitations and incomplete data mining, some authors’ findings were not included in this review and we apologize for that. Given the high dependence of the IAV life cycle on the host environment, additional, as-yet undiscovered interacting host proteins must exist. Therefore, new host proteins remain to be discovered, and the molecular details and mechanisms of how they influence the replication, pathogenesis, and transmissibility of IAV will have to be unveiled. These data may provide valuable information for the risk warning and formulation of control measures against animal and human influenza. As such knowledge continues to accumulate, the design and development of novel antiviral drugs and better vaccines will undoubtedly follow.

## Declaration of competing interest

The authors declare no competing financial interests.
